# A Case of Tænia Solium Causing Herpes

**Published:** 1840

**Authors:** John M. Bigelow


### II. *A case of Tænia Solium, apparently causing Herpes.* By J. M. Bigelow, M. D.

Mrs. D——, aged about thirty years, of a complexion so light as almost to be denominated an albino, consulted me in August, 1837, for an obstinate herpetic eruption on the hands and fore-arms. She stated to me, that, about five years before, in the winter, the herpetic complaint first made its appearance at the roots of the nails of each hand. It left her in the spring, and did not re-appear until the succeeding winter, when it was more severe and spread higher up than in the preceding winter. It thus continued to augment in severity, duration and size, until the summer of 1837, when it did not leave her, but spread above the elbows. The nails of the fingers on each hand had been removed several times by it. The thumb nails, however, had not been touched. The interval of the disease in the summer of 1836, had been very short, and, as before stated, it had continued uninterrupted, although mitigated in severity, through the summer of 1837. She had unsuccessfully tried the prescriptions of several physicians; indeed, the application of external medicaments invariably had the effect to aggravate the disease. When I first saw the patient in August, I prescribed the hydro-sulphuret of antimony, in union with the white oxyde of arsenic, to be taken internally in small and alterative doses, and the use of the chlorate of soda, as an alternate lotion. This plan was perseveringly pursued for a considerable length of time, without any apparently beneficial effects whatever. The 20th of September the disease broke out with as much severity as in any preceding year. At this time she stated to me that she had symptoms of tape worm since February last. The only symptoms were their occasional appearance in the evacuations of the bowels. I now prescribed the polypodium filix mas, followed by a brisk cathartic of calomel and gamboge, upon the principle of Madame Nouffleur. A short persistence in this course brought away, in the language of my patient, "immense quantities" of *tænia solium*, from single joints to detached portions of the length of five or six feet. At the time I prescribed the anthelmentic course, I recommended my patient to abstain from all applications to the herpetic eruption. She only made use of sweet cream to soothe the smarting and irritation of the sore. The herpetic disease began to amend immediately, and in less than a month was healed entirely.

November 11th.—She still had symptoms of worms, but no herpetic eruption—prescribed cathartic pills and oil of worm seed.

31st December.—*Tænia* still continue to be occasionally evacuated, and the eruption had returned, but in a much slighter degree than for several winters past. Prescribed the plan adopted by Madame Nouffleur again, to be followed by the use of carbonate of iron, in moderate doses, for some time. My patient living a considerable distance from town, I have not since heard from her.

☞ The principal facts of the above case were communicated to Dr. S. E. Evans, of Transylvania University, and embodied into his Inaugural Thesis.

